# On development of functional brain connectivity in the young brain

**DOI:** 10.3389/fnhum.2013.00650

**Published:** 2013-10-08

**Authors:** G. E. Anna-Jasmijn Hoff, M. P. Van den Heuvel, Manon J. N. L. Benders, Karina J. Kersbergen, L. S. De Vries

**Affiliations:** ^1^Department of Neonatology, Wilhelmina Children's Hospital, University Medical Center UtrechtUtrecht, Netherlands; ^2^Department of Psychiatry, Brain Center Rudolf Magnus, University Medical Center UtrechtUtrecht, Netherlands

**Keywords:** resting-state functional MRI, functional connectivity, brain development

## Abstract

Our brain is a complex network of structurally and functionally interconnected regions, shaped to efficiently process and integrate information. The development from a brain equipped with basic functionalities to an efficient network facilitating complex behavior starts during gestation and continues into adulthood. Resting-state functional MRI (rs-fMRI) enables the examination of developmental aspects of functional connectivity (FC) and functional brain networks. This review will discuss changes observed in the developing brain on the level of network FC from a gestational age of 20 weeks onwards. We discuss findings of resting-state fMRI studies showing that functional network development starts during gestation, creating a foundation for each of the resting-state networks (RSNs) to be established. Visual and sensorimotor areas are reported to develop first, with other networks, at different rates, increasing both in network connectivity and size over time. Reaching childhood, marked fine-tuning and specialization takes place in the regions necessary for higher-order cognitive functions.

## Introduction

**Figure 1:**
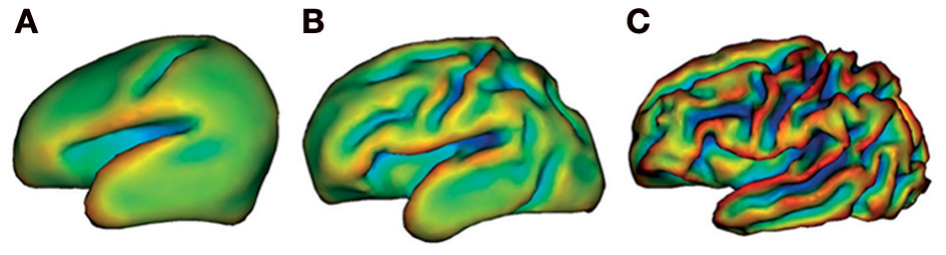
**Cortical folding at 26 weeks (A), 29 weeks (B), and 36 weeks gestational age (C).** This illustrates considerable macroscopic changes of the developing preterm brain. Images are generated by brain surface rendering with a mathematical morphology approach. The different colors delineate surface curvature. Reproduced with permission from Elsevier (Ment et al., 2009).

The change from basic behavioral patterns during the first months after birth to being able to reason logically as an adult illustrates that development of the brain with age is very important. Although these cognitive manifestations of brain development are impressive, brain maturation may be even better appreciated by the macroscopic anatomical changes which the brain undergoes before birth. Considerable increases in both cortical folding and volume have been studied from 26 weeks gestational age (Figure 1) (Dubois et al., 2008; Ment et al., 2009). Even though this process continues also beyond the age of 2, important changes in cortical folding and volume are observed before 2 years of age. From the age of 2 onwards, both cognitive and behavioral development becomes more prominent, while the extent of macroscopic anatomical changes and myelination is fairly limited compared to changes before the age of 2 (Paus et al., 1999). Thus, development of these domains during late human development is more likely to rely on microstructural or functional changes (Yakovlev and Lecours, 1967; Paus et al., 1999).

**Figure 2:**
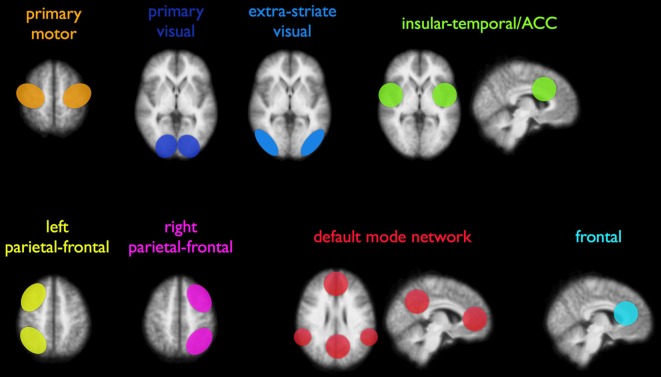
**Illustration of eight commonly reported resting-state networks (RSNs), consisting of distant areas that show functional coupling.** Both unilateral and bilateral connectivity networks have been consistently reported in adults. Reproduced with permission from Elsevier (Van den Heuvel and Hulshoff Pol, 2010).

For studying brain development, previous research has shed light on different aspects of the early developing brain, such as cerebral volume, cortical morphology, gray/white matter ratios, and brain metabolism (Chugani, 1998; Dubois et al., 2008; Hüppi, 2011). Studies have revealed several aspects of brain structure and function using techniques such as conventional MRI, diffusion-tensor imaging (DTI), positron emission tomography (PET), and electroencephalography (EEG) (Chugani, 1998; Smit et al., 2012; Vasung et al., 2013). An imaging technique to study the functional interactions between brain regions is resting-state functional MRI (rs-fMRI), which measures the level of correlation between endogenous brain signals. Evidence is emerging that this spontaneous activity is predominantly of a neuronal origin and can thus reflect functional connectivity (FC) within the brain (Fox and Raichle, 2007; Leopold and Maier, 2011). In case of a significant overlap of spontaneous activation patterns of two spatially distant brain regions, a level of FC is assumed and a so-called resting-state network (RSN) can be identified (Fox et al., 2005; Fox and Raichle, 2007). At least eight resting-state networks, among others motor, visual, attentional, and default-mode networks, have been described in adult humans (Figure 2) (Damoiseaux et al., 2006; Smith et al., 2009; Van den Heuvel and Hulshoff Pol, 2010). Also animal studies in rodents (Becerra et al., 2011; Jonckers et al., 2011) and monkeys (Hutchison et al., 2011; Mars et al., 2011) have shown analogous large-scale brain networks, validating concepts of functional network connectivity in humans.

This review will address some of the developmental changes that can be observed in the functional networks of the young brain using rs-fMRI. First, to illustrate how the early human brain develops qualitatively, connectivity patterns of preterm and full-term children up to the age of 2 will be discussed. After a concise description of late human brain development, i.e. from 2 years of age onwards, the focus will shift toward quantitative measurements of brain development. Lastly, a brief summary, a discussion of a number of technical limitations of rs-fMRI as well as future directions for research will be provided.

## Early human development

**Figure 3:**
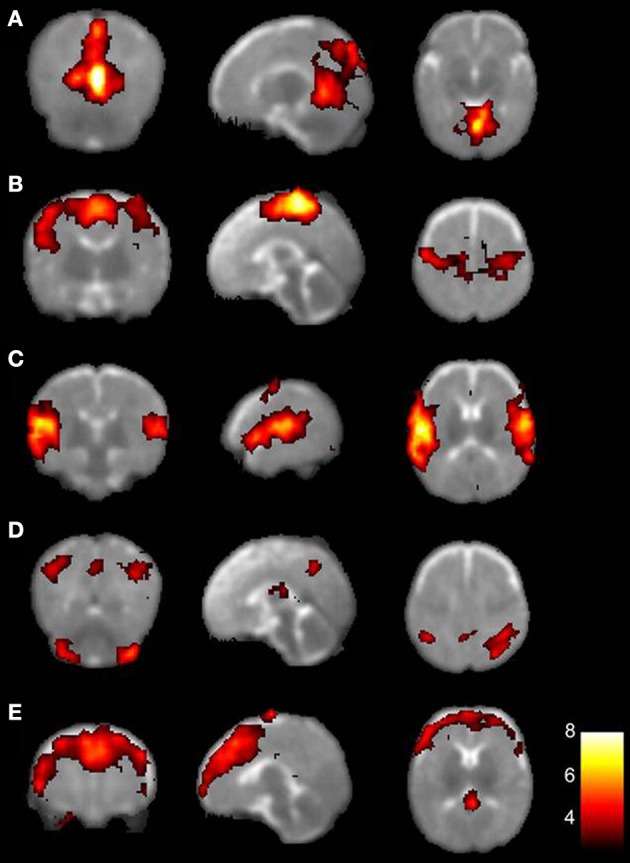
**The five resting-state networks consistently found in preterm infants.** Each row depicts one resting-state network represented on an axial T2-weighted template, with on the left hand side the left hemisphere. Colors indicate correlation strength, with increasing correlation strength toward the yellow part of the spectrum. **(A)** primary visual areas; **(B)** bilateral somatosensory and motor cortices; **(C)** bilateral temporal/inferior parietal cortex encompassing the primary auditory cortex; **(D)** posterior lateral and midline parts of the parietal cortex and lateral aspects of the cerebellum; **(E)** medial and lateral sections of the anterior prefrontal cortex. With permission reproduced from (Fransson et al., 2007). Copyright National Academy of Sciences, U.S.A. (2007).

In recent years, early brain maturation has been studied by examining the resting-state dynamics in both prenatal and postnatal life. Recently, two studies have conducted fetal rs-fMRI showing that it may be possible to map FC of healthy fetuses (Schöpf et al., 2012; Thomason et al., 2013). Almost half of all bilateral functional networks could be identified *in utero* from 24 weeks gestational age onwards, with increasing connectivity strength toward full-term age (Thomason et al., 2013). Similar maturation effects have been observed in prematurely born infants (Fransson et al., 2007; Doria et al., 2010; Smyser et al., 2010). Five functional networks were indentified in extremely preterm to early preterm infants at term-equivalent age, encompassing the primary visual cortex, bilateral sensorimotor area, bilateral auditory cortex, precuneus area, lateral parietal cortex, cerebellum, and the medial and dorsolateral prefrontal cortex (Figure 3) (Fransson et al., 2007). Additionally, default-mode and executive control RSNs have been reported in very preterm to late preterm infants as well (Doria et al., 2010). One network in the prematurely born infants (Figure 3, network **D**) could not be directly linked to a comparable network known in adults. Also, immaturity of the identified networks was characterized by less extension into the posterior-anterior direction compared to adult networks. Although contradicting results have been reported on lateralization of developing networks in which also unilateral networks have been described (Liu et al., 2008), more evidence seems to be present for bilateral FC patterns, already present at the neonatal stage (Fransson et al., 2007, 2009; Lin et al., 2008; Kelly et al., 2009; Gao et al., 2013). Based on the consistent findings of both fetal and neonatal rs-fMRI studies it can be hypothesized that the foundations of resting-state networks are already laid down before term age, with rapid neural growth in the last trimester of pregnancy (Doria et al., 2010).

The default-mode network (DMN) is a network that received considerable attention in FC studies in children. The adult DMN encompasses the posterior cingulate cortex (PCC), the precuneus, the medial prefrontal cortex (mPFC), the orbital frontal gyrus, the anterior cingulate cortex (ACC), the inferolateral temporal cortex, the parahippocampal gyrus as well as the bilateral parietal cortex (Raichle et al., 2001; Thomason et al., 2008; Van den Heuvel et al., 2009; Damaraju et al., 2010). Which exact collection of functions the network employs in humans has not yet been elucidated. However, the DMN is considered to be important for internally focused tasks, such as episodic memory, self-referential thought, and other social cognitive processes (Buckner et al., 2008; Uddin et al., 2010). Resting-state studies that could not detect a DMN in preterm infants have suggested the existence of a pre-DMN, or “proto-DMN” (Fransson et al., 2007, 2009, 2011; Doria et al., 2010; Power et al., 2010; Smyser et al., 2010). This network, composed of the bilateral parietal cortex and the precuneus/PCC, has been suggested to be a fragment of the posterior part of the adult DMN, forming the basics of the DMN at term age (Doria et al., 2010).

Even though the frameworks of the DMN and other RSNs can be recognized at term age, some networks appear to be more developed than others. For example, FC of the visual and auditory networks is relatively mature compared to other networks in preterm infants around 36 weeks of gestation (Lin et al., 2008; Doria et al., 2010). Medial regions develop through different connectivity patterns as compared to lateral regions (Smyser et al., 2010). In these medial regions, such as in the anterior cingulate, an increase in interhemispheric connectivity can already be detected as early as 26 weeks post-menstrual age (PMA) (Smyser et al., 2010; Thomason et al., 2013). Seeds in lateral brain regions, for instance in the sensorimotor cortex, have connections that extend over a relatively large distance to their homotopic counterparts. Interhemispheric connectivity between these laterally located areas still cannot be detected by 38 weeks PMA (Smyser et al., 2010). They appear to first intensify local connection strength before the connection toward the homotopic counterpart is established (Smyser et al., 2010). Increasingly coherent interhemispheric activity and high thalamic FC during the period of accelerated neural development demonstrates the critical importance of this last period of pregnancy for brain network maturation (Smyser et al., 2010, 2011; Uddin et al., 2010; Thomason et al., 2013).

### The first years of life

Next to different developmental rates of connectivity strength, development of network size appears to differ between networks as well. Changes in network size, represented by a percentage of brain volume, have been observed during the first years of life. Comparing infants of 2 weeks with infants at 2 years of age, several RSNs have shown to exhibit a significant increase in FC as well as cerebral volumes of cortical connectivity (Lin et al., 2008). However, the latter study showed that growth of network volume is not necessarily associated with a simultaneous increase in FC strength across all RSNs. Whereas FC strength of the sensorimotor RSNs is comparable to the visual RSNs, the sensorimotor network exhibits the most significant increase in volume between 2 weeks and 1 year, preceding the growth of the visual network, which occurs predominantly between 1 and 2 years of age (Lin et al., 2008). Lack of linear correlation between increases in connectivity strength and network size also applies to the DMN in the first 2 years (Gao et al., 2009). With a gradual increase in network size and a decrease in fragmentation during this period, it is possible that the more fragmented state of the DMN early in development could be a sign of immaturity, in which the network achieves adult-like properties around 1 year of age (Fair et al., 2008; Gao et al., 2009; de Bie et al., 2011). The dorsal attention network synchronizes in a similar way, although its configuration also seems to be influenced by network-network interactions with the DMN (Gao et al., 2013).

## Late human development

From the age of 2 years onwards, neurodevelopment is characterized by a gain in higher-order cognitive abilities, such as attention and memory. Networks supporting these abilities show differences between children and adults, which is assumed to reflect a process of maturation. For example, EEG in healthy 5–8-year-old children has demonstrated that RSNs associated with higher-order cognitive functions, such as the DMN, cingulo-opercular, ventral, and dorsal frontoparietal RSNs, still have a primitive architecture compared to adults (de Bie et al., 2011). Characteristics of such a primitive architecture are lower overall FC, weak within-network connectivity and presence of aberrant connections between distant brain areas as compared to adults. The sensorimotor area, which starts its development relatively early (de Bie et al., 2011), did not show these immature characteristics. Rather, FC of the sensorimotor area, which is similar to adults, may suggest mature-like characteristics of primary networks by the age of 7 (de Bie et al., 2011). As for the DMN, a more integrated network is found in teenagers and young adults (Fair et al., 2008, 2009). By the age of 10, each of the major regions of the DMN are present, with areas in the mPFC, the PCC, the left and right medial temporal lobe and the left and right angular gyrus (Supekar et al., 2010). Small spatial differences from adult patterns persist and FC of all RSNs, including the DMN, is still reduced. At 12 years of age, overall FC as well as network size is still decreased in comparison with adults (Jolles et al., 2011). However, areas associated with higher cognitive and emotional processing (for instance the executive control system, the dorsal attention system and the DMN) showed locally increased FC compared to the level of connectivity in these areas in adults. Hence, the basic configuration of RSNs may be subject to fine-tuning and specialization during the first years of adolescence, especially in the regions necessary for higher-order cognitive functions. Some studies have suggested that brain maturation may be reflected by a decrease in connectivity of short-range links and an increase of FC of long-range connections (Fair et al., 2007, 2008; Kelly et al., 2009; Supekar et al., 2009). Yet, caution is warranted regarding the interpretation of the results, as observed effects may at least be partly explained by effects of head motion, which has been shown to impose systemic effects on rs-fMRI measures (Power et al., 2012; Van Dijk et al., 2012).

## Quantitative measurements of brain development: graph theory

Graph theory describes and quantifies complex whole-brain networks (for a comprehensive review of graph theory, see Bullmore and Sporns, 2009), which allows interpretation of different quantitative measurements into qualitative aspects of whole-brain organization.

Initially, communication between networks seems to be mostly localized to areas in close anatomical proximity (Fair et al., 2007, 2009; Supekar et al., 2009; Gao et al., 2011). During development, large-scale brain networks transform from a locally oriented organization to a more integrated topology (Fair et al., 2007, 2009; Supekar et al., 2009; Gao et al., 2011). The presence of “functional hubs” is an example of how graph theoretical measures may provide insight into functional cerebral architecture. Functional hubs, which are brain areas with a high FC density, are thought to be important for efficient neural signaling and integration of information (Buckner et al., 2009; Tomasi and Volkow, 2011). Cortical hubs and their related cortical networks in healthy, full-term infants have been found to be bilaterally connected and mainly located in the homomodal primary sensorimotor, visual, and auditory brain regions (Fransson et al., 2011). As of the age of 2, the posterior cingulate cortex/retrosplenial (PCC/Rsp) connection exhibits considerable strength, which would make it suitable to function as a primary cortical hub within the developing DMN (Gao et al., 2009). With age, FC between hub and non-hub connections increases strongly, while connectivity between hubs remains relatively stable (Hwang et al., 2013).

## Methodological considerations

Currently, the approaches most commonly used for the analysis of the rs-fMRI data are seed-based or region-of-interest (ROI) analyses, independent component analysis (ICA) and graph theory. The latter approach is used for describing properties of the functional connections rather than establishing them; some applications of graph theory have been discussed in the previous section. In ROI-based analysis the time series of a brain region of interest are correlated to time series of other brain regions. The functional connections of this predefined area of the brain, or seed, can thus be determined. Its relative simplicity may be a disadvantage, as whole-brain connectivity patterns—without predefined brain regions to correlate to—cannot be evaluated. Also, it can be more difficult to detect novel links (Gao et al., 2009). Alternatively, this limitation does not apply to ICA, which enables formation of a whole-brain connectivity map. Although an a priori hypothesis is not necessary to run the statistics, the interpretation of the analysis is more difficult than the ROI-based analysis method (Fox and Raichle, 2007; de Bie et al., 2011). When further comparing seed-based analysis and ICA, the resting-state data results appear to correspond fairly well (Gao et al., 2011; Rosazza and Minati, 2011). Yet, the level of correspondence slightly decreases when more components are included in the analysis (Rosazza and Minati, 2011). The application of different preprocessing steps enhances the heterogeneity among rs-fMRI studies, further complicating comparison and uniform interpretation of rs-fMRI studies (Lee et al., 2013). In addition, rs-fMRI data may also be subject to other confounding effects possibly leading to misinterpretation. Potential and unknown effects of other neurophysiological processes or sedation on the rs-fMRI data (Nallasamy and Tsao, 2011; Birn, 2012), as well as the already mentioned sensitivity to effects of motion (Power et al., 2012; Van Dijk et al., 2012), with reported intra-individual differences (Honey et al., 2009) could impinge on reproducibility and question the neuronal nature of observed developmental effects.

### Effects of prematurity on FC

Prematurity, especially in the context of a complicated postnatal course, may have adverse effects on gray matter volume, myelination, cerebral surface area, and overall cerebral volume (Hüppi et al., 1996; Inder et al., 2005; Kapellou et al., 2006). However, data on effects of prematurity on FC development are still limited. The networks identified in preterm infants have been reproduced in healthy full-term neonates, supporting at least similar RSN architecture (Fransson et al., 2009). In addition, despite small differences in the basal ganglia, the visual cortex and the cerebellum, no major differences have been reported between the anatomical locations of RSNs in preterm infants without serious postnatal complications and full-term controls (Damaraju et al., 2010; Doria et al., 2010; Smyser et al., 2010). The studies cited included prematurely born infants without acquired brain injury or early developmental problems and provided limited information on long-term neurodevelopmental outcome. Therefore, to what extent brain development of preterm infants may be different from full-term neonates warrants further exploration. Prematurely born infants with only minor cerebral abnormalities showed disruption in network architecture, especially in thalamo-cortical connections (Smyser et al., 2010). Compared to the networks of the full-term infant, preterm infants scanned at term-equivalent age had lower correlations and less connectivity between lateral seeds. Moreover, whereas infant born at term showed characteristics of a coherent network with possible DMN precursors, term-equivalent premature infants did not (Smyser et al., 2010). Follow-up data of preterm infants at 36 months of age showed lower overall connectivity compared to full-term peers (Damaraju et al., 2010). Long-term effects of premature birth on FC have been observed in young adulthood, in which alternative functional circuits involved in language have been described (Constable et al., 2013).

Summarizing, a number of studies have looked at resting-state dynamics in premature populations, but only limited data is available on possible effects of prematurity on FC development. More studies in both preterm and healthy term infants with long-term follow-up are therefore required to improve insights into brain development.

## Directions for future research

All of the aspects outlined in the previous section should be taken into consideration in the interpretation of developmental effects in rs-fMRI studies and also provide directions for further investigations. In addition, elucidating the structure-function relationship of networks by further combination of imaging techniques could improve insights into the mechanisms behind functional network development. Similar maturational effects can be observed with DTI measuring structural parameters (Vasung et al., 2013) and arterial spin labeling MRI to map perfusion (De Vis et al., 2013). Furthermore, the translational aspect of developmental connectivity patterns toward executive functions and behavior merits attention as well, which ultimately may improve prediction of neurodevelopmental outcome or disease progression by rs-fMRI techniques. Considering that the last trimester of pregnancy may be especially relevant for adequate FC development (Doria et al., 2010), studying effects of prematurity related to both brain maturation and neurocognitive outcome could be an important application. Full-term infants with neonatal encephalopathy, in whom prediction of cognitive outcome is often difficult, may also be a relevant study population. In addition, a possible effect of gender on RSN patterns has been suggested (Biswal et al., 2010; Weissman-Fogel et al., 2010; Zuo et al., 2010; Gong et al., 2011). Structural MRI studies have shown that in particular across puberty gradual sexual dimorphisms develop, with unknown relationships between gender, puberty and neural development (Blakemore, 2011). So far, only one study addressed gender differences in functional homotopy in children (Zuo et al., 2010). Therefore, to what extent gender effects might influence developmental patterns of FC in neonates, children or young adults remains unknown. Lastly, this review supports the notion that foundations of each of the RSNs are laid down before term age, after which fine-tuning and specialization of these networks take place. It has been suggested that a genetic substrate for functional networks may exist (e.g., Glahn et al., 2010). Twin-studies in children and adults indeed show significant effects of heritability, primarily on the level of whole-brain connectivity efficiency and functional organization (Fornito et al., 2011; Van den Heuvel et al., 2013). More research into the genetic control underlying functional network organization is needed, also toward possible identification of specific deficits in FC relevant for neuropsychiatric diseases, such as autism or schizophrenia.

## Conclusion

This review described developmental changes observed in the functional networks of the brain from 20 weeks of gestation onwards using rs-fMRI. Even though the techniques used to acquire and analyze rs-fMRI data leave room for improvement, all of the efforts so far have led to significant insights into brain development. Even before term age, a network with foundations of each of the RSNs can be recognized (Doria et al., 2010; Fransson et al., 2011). RSNs differ in their growth trajectories, but fine-tuning and specialization of RSNs is generally characterized by increasing FC, network volume, and coherence. The next step in developmental fMRI research may be to explore the origins of inter-individual network variation as well as associations with cognitive functioning and behavior by combining structure and function at different ages, in both healthy and diseased states.

### Conflict of interest statement

The authors declare that the research was conducted in the absence of any commercial or financial relationships that could be construed as a potential conflict of interest.
